# Light‐driven DNA repair at atomic and picosecond resolution revealed via time‐resolved serial femtosecond crystallography

**DOI:** 10.1002/ctm2.1566

**Published:** 2024-01-27

**Authors:** Yuhei Hosokawa, Manuel Maestre‐Reyna

**Affiliations:** ^1^ Institute of Biological Chemistry Academia Sinica Taipei Taiwan; ^2^ Department of Chemistry National Taiwan University Institute of Biological Chemistry Academia Sinica Taipei Taiwan

1

Genome integrity is threatened by solar ultraviolet (UV) light, which reacts with adjacent DNA pyrimidine bases to produce undesirable covalent bonds. As the most common UV‐induced lesion, cyclobutane pyrimidine dimers (CPDs) have been strongly linked to cancer. For example, patients with xeroderma pigmentosum, a genetic defect in DNA repair, are at increased risk of skin cancer.^1^ To combat CPD, Human beings use a complex enzymatic pathway called nucleotide excision repair (NER). However, most other organisms employ a much simpler and more effective mechanism where a single protein, CPD photolyase, recognises and repairs the CPD lesion.^2^ CPD photolyases are extremely efficient, because they use the energy of solar blue light to repair UV‐photolesions. Because of the simplicity and speed of the reaction by CPD photolyase, the clinical application of CPD photolyase has been thoroughly explored.^3^

Apart from the therapeutic potential of CPD photolyase, studying its repair process at the atomic level is of great biological interest, as it explains how most organisms protect themselves from DNA damage. The key reaction of CPD photolyase is initiated by exposing its catalytic cofactor, flavin adenine dinucleotide in its fully reduced state (FADH^−^), to blue light. In its light‐excited state, FADH^−^ injects an electron into the CPD lesion, which leads to two undesired covalent bonds breaking within the CPD, turning the photolesion into two normal nucleobases (Figure 1). Recently, we and Christou et al. independently and successfully visualised the entire photolyase reaction cycle in real time and at atomic resolution using time‐resolved serial femtosecond crystallography (TR‐SFX).^4^, ^5^ The technique relies on high‐energy X‐ray sources called X‐ray free electron lasers (XFELs),^6^ in which experimental data can be collected faster than chemical reactions take place. By combining faster‐than‐chemistry data collection with a pump laser system to initiate photolyase‐catalysed DNA repair, we were able to produce a “molecular movie” revealing the conformational changes during the repair reaction itself, and also during the post‐repair process. In the following section, we provide a brief overview of our movie.

**FIGURE 1:**
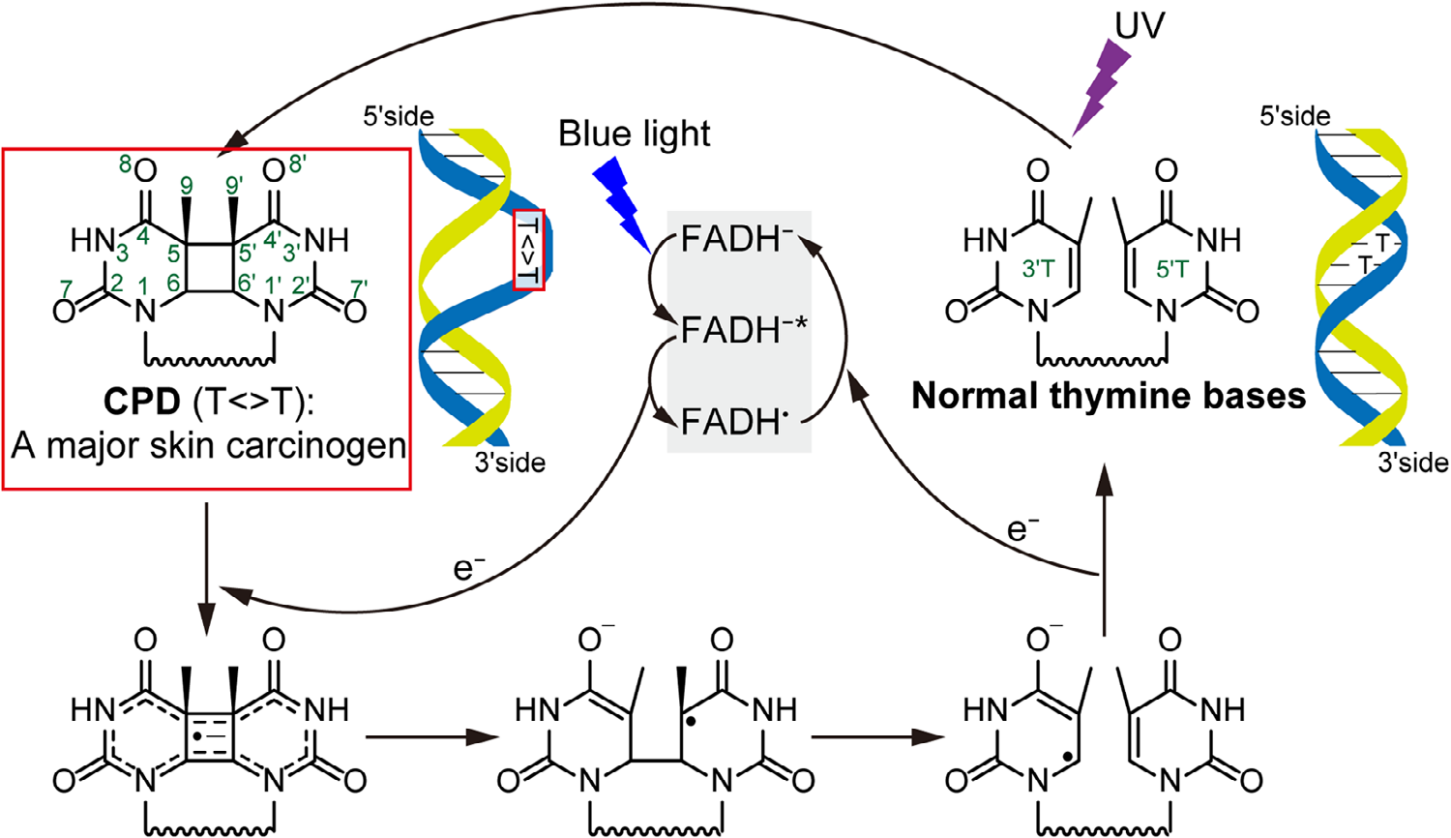
Schematic overview of the formation and repair of CPD. CPD is formed by UV light irradiation to adjacent pyrimidine bases (e.g., Thymine bases) in DNA and is a major skin carcinogen. CPD photolyase repairs CPD by injecting an electron from its photoexcited FAD cofactor (FADH^−*^) to CPD. CPD, cyclobutane pyrimidine dimer; FADH, flavin adenine dinucleotide; UV, ultraviolet.

Our test subject was the archaeal *Methanosarcina mazei* class II CPD photolyase (*Mm*CPDII). In previous studies, we were able to analyse the FAD conformational changes in *Mm*CPDII upon photoreduction, the process by which the enzyme renders its FAD cofactor active.^7^, ^8^ After photoreduction, the oxidised, inactive, and planar FAD transforms into the reduced, active FADH^−^. In our previous study, we discovered that while *Mm*CPDII‐bound FAD is planar, turning into FADH^−^ causes a pronounced V‐shape, and thus we knew that we could use the FAD geometry to follow electron flow during the repair reaction (Figure 2A). For example, 100–250 ps after illumination of FADH^−^ in the presence of a CPD lesion, the cofactor swung, indicating the presence of the photoexcited FADH^−*^ state. By 450 ps after illumination, FAD had changed its geometry again, becoming planar due to partial oxidation into the radical FADH**^•^** state, and suggesting that electron injection into to the CPD substrate was mostly done at this point (Figure 2A). Then, about 2–6 ns after illumination, FADH**^•^** again became more V‐shaped (Figure 2A), suggesting that the electron had returned after accomplishing the bond rearrangement of CPD, that is, DNA repair (Figure 2A).

**FIGURE 2:**
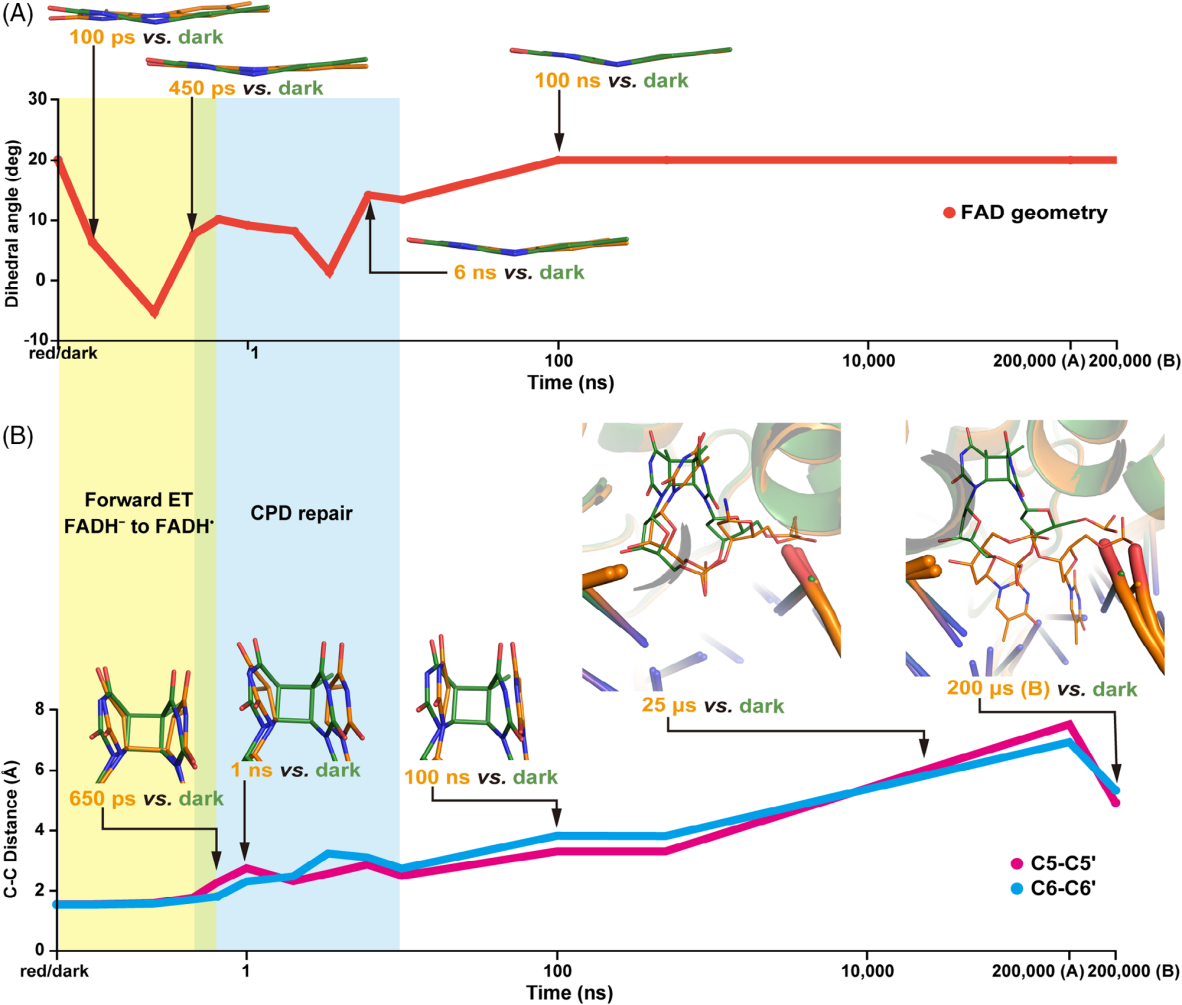
The progress in DNA repair by CPD photolyase. The representative structures of (A) FAD and (B) CPD after illumination (orange) are superposed over the dark structure (green). (A) Time trace of a dihedral angle describing FAD geometry (red line). (B) The structural changes around CPD are highlighted by the time trace of the average C5–C5’ and C6–C6’ distances, as shown by magenta and cyan lines, respectively. CPD, cyclobutane pyrimidine dimer; FADH, flavin adenine dinucleotide.

Simultaneously to monitoring electron flow via the FAD geometry, we could also directly observe CPD transformation into repaired DNA. Here, by 650 ps, the first bond‐breaking event had taken place (Figure 2B). Additionally, we could observe how the *Mm*CPDII protein stabilised this highly reactive intermediate species via targeted electrostatic contacts and solvent rearrangements. Finally, by 1 ns after illumination, the second bond broke, yielding the repaired DNA after electron return (Figure 2B). These results agree very well with previous spectroscopic studies,^9^, ^10^ which suggested that the CPD bond rearrangement occurred within a few nanoseconds after illumination. However, such studies were limited in their analysis to the status of only the CPD, or the FAD, whereas our molecular movie captured the conformational and chemical changes of every single actor in the reaction, including not only FAD and CPD, but also the protein and the remainder of the DNA.

For example, the TR‐SFX experiments not only clearly captured CPD bond‐breakage, but also the active site recovery and the release of the repaired product from the active site (Figure 2B), which are mostly spectroscopically silent, and therefore undetectable via prior methods. Surprisingly, here we found out that although the photolyase reaction cycle is finished within a few nanoseconds, a complete recovery of the FADH^−^ and protein active site geometries, as observed in the pre‐illumination state, took hundreds of nanoseconds. Further, at this time, the repaired thymine bases were still inside the active site and fully stacked to each other, suggesting that *Mm*CPDII does not rush to the product release (Figure 2). Only after tens of microseconds did the bases start to exit the active site, displacing several protein–DNA interactions keeping the complex together, the so‐called bubble‐intruding region (BIR), and thus coordinating repair, enzyme recovery and complex release in an ordered sequence of events.

In conclusion, our TR‐SFX measurement successfully visualised the full sequence of events leading to repair of UV‐induced DNA damage by a photolyase. For the first time, we were able to show the full reaction cycle of an enzyme, from activation and chemical transformation to product release. Importantly, we produced highly detailed structures of the short‐lived reaction intermediates, which not only helped us to understand DNA repair mechanisms but also may inspire chemists in the development of DNA‐protecting drugs, or artificial DNA repairing systems.

## AUTHOR CONTRIBUTIONS

YH and MMR wrote the manuscript. YH and MMR performed experiments and analyzed data. MMR conceived research.

## ETHICS STATEMENT

The authors declare no competing interests.
